# Effect of exercise intervention on anxiety among college students: a meta-analysis

**DOI:** 10.3389/fpsyg.2025.1536295

**Published:** 2025-03-14

**Authors:** Peng Chen, Nur Shakila Mazalan, Denise Koh, Yusha Gu

**Affiliations:** ^1^Faculty of Education, National University of Malaysia, Bangi, Selangor, Malaysia; ^2^Department of Sports Studies, Faculty of Physical Educations, Ningxia Normal University, Guyuan, China

**Keywords:** exercise, anxiety, college students, mental health, effect size

## Abstract

**Objective:**

This study conducted a meta-analysis to examine the impact of exercise interventions on anxiety levels among college students.

**Methods:**

Under Review Manager 5.3 and Stata17.0, subgroup analyses were conducted on data from 514 subjects across 10 studies from Chinese and English literature. The analyses examined intervention content, intervention period, single intervention duration and intervention frequency. A random effects model was employed to assess the overall effect size and heterogeneity.

**Results:**

The exercise intervention demonstrated a significant effect on reducing anxiety among college students, with a large effect size (*d* = −0.83). The heterogeneity test of intervention content (I^2^ = 0%) revealed high consistency in the specific content of exercise intervention across studies. However, substantial heterogeneity was observed in single intervention time (I^2^ = 75%), intervention frequency (I^2^ = 75%), and intervention period (I^2^ = 72%), indicating significant variations across studies. These differences suggest that varying durations, frequencies, and periods of intervention yielded different effects on college students’ anxiety levels.

**Conclusion:**

This meta-analysis has found that structured physical exercise programs, especially mind–body integrative exercises, can alleviate anxiety to the greatest extent, offering evidence-based guidance for implementing targeted exercise interventions in college mental health programs.

**Systematic review registration:**

https://doi.org/10.37766/inplasy2024.11.0006.

## Introduction

Anxiety is a complex emotional state, characterized by worry, restlessness, and fear about the future (Rowa et al., 2017). Psychologically, anxious individuals exhibit heightened attention to potential negative events, often leading to unfounded or exaggerated worries (Craske et al., 2011). This condition manifests through various physiological responses, including rapid heartbeat, accelerated breathing, increased sweating, and muscle tension (Hyman and Pedrick, 2012). Studies indicate that the incidence of anxiety among college students reaches 54.4%, representing one of their most prevalent psychological challenges (Lun et al., 2018). The impact of anxiety extends beyond psychological distress, affecting the autonomic nervous system (ANS) through disruption of both sympathetic and parasympathetic functions (Chalmers et al., 2014). Chronic anxiety can compromise the body’s immune system, elevating disease susceptibility (Dantzer, 2018).

Recent meta-analysis have explored various exercise interventions for anxiety management in college students. Du et al. (2022) analyzed 12 studies involving 1,000 participants, finding that Tai Chi therapy significantly reduced anxiety symptoms, particularly when combined with mindfulness interventions and structured as thrice-weekly, 80–90-min sessions over 12 weeks. Song et al. (2021) examined 44 studies, revealing the effectiveness of aerobic exercise, traditional Chinese exercise, and meditation in alleviating depressive symptoms, with aerobic exercise showing superior outcomes for anxiety. Lin and Gao (2023) evaluated nine studies, demonstrating that moderate to high-intensity exercise with longer duration and higher frequency significantly improved anxiety symptoms.

Subsequently, a meticulous dissection of the idiosyncratic contributions and innovative insights advanced by this research will be carried out. To commence with, when it comes to the pinpointed focus of the research object, prior meta-analyses, albeit encompassing research on exercise interventions for college students, exhibited a relatively wide–ranging scope. In contrast, this study precisely zeroes in on the impact of mind–body integrated exercises, such as yoga, qigong, taijiquan, and Pilates, on the anxiety levels of college students. By taking these as the core research subjects, it fills the gap in the sub-categorization of exercise types in previous studies, providing more targeted references for exercise selection in college student anxiety interventions. Mind–body exercise refers to a category of physical activities that place a particular emphasis on the connection and interaction between the mind and the body (Kim, 2000). It combines physical movement with mental focus, incorporating elements such as mindfulness, meditation, and specific breathing techniques, aiming to enhance physical and mental health, reduce stress, and boost self-awareness (Gard et al., 2014). There are several differences between it and conventional sports: In terms of focus, mind–body exercise takes both the mind and body into account (Mehling et al., 2011). For example, yoga emphasizes focusing on the breath and being present in the moment (Salmon et al., 2009), while conventional sports mainly focus on physical performance (Durand-Bush and Salmela, 2002). For instance, running pursues speed and endurance. Regarding movement characteristics, mind–body exercise features slow, smooth, and controlled movements (La Forge, 2005). Just like the gentle and continuous movements in Tai Chi (Lan et al., 2002), conventional sports are more diverse, including quick and explosive movements (Suchomel et al., 2018). In terms of skill acquisition, mind–body exercise focuses on cultivating the abilities of mental concentration, breath control, and coordination between the body and mind, which requires long-term patient practice (Aposhyan, 2018). Conventional sports mainly involve mastering specific sports techniques, such as running postures and swimming strokes (Launder and Piltz, 2013).

Furthermore, the merit of comprehensively comparing multiple exercise modalities is manifested in the fact that previous research predominantly concentrated on the influence of a solitary exercise modality on anxiety, or merely made cursory references to a handful of exercise modalities, falling short of conducting a thorough comparative analysis. This study innovatively incorporates multiple mind–body integrated exercises into the same research framework, systematically comparing their effects on alleviating anxiety among college students. Through this comprehensive comparison, the advantages and disadvantages of different mind–body integrated exercises in reducing college students’ anxiety levels can be clearly presented, offering a wider array of intervention strategies for university mental health programs and healthcare providers. Finally, as for the highlight of in-depth integration of exercise parameters, although previous studies did refer to the parameters of exercise interventions, most were specific parameter studies for a single exercise, lacking an integrated analysis of parameters such as intervention duration, frequency, and intensity across multiple exercises. However, this study conducts a comprehensive analysis of these key parameters of multiple mind–body integrated exercises through subgroup analysis, aiming to identify the optimal parameter combinations suitable for different mind–body integrated exercises, thus providing a scientific basis for formulating personalized and precise exercise intervention programs. This represents a significant expansion of existing research on exercise intervention parameters.

This study adopts the method of meta-analysis, with a focus on exploring the impact of mind–body integrated exercise interventions on the anxiety levels of college students, aiming to synthesize data on intervention duration, frequency, and intensity across multiple exercise modalities. Given the limited understanding of how these exercise-based interventions precisely affect college students’ anxiety, and the lack of clear insights into which exercise types and parameters are most effective, the following research hypotheses are proposed.

*Hypothesis* 1: Exercise has a significant effect in alleviating the anxiety of college students.

*Hypothesis* 2: Among all forms of exercise, Taijiquan demonstrates the most remarkable efficacy in reducing the anxiety levels of college students.

*Hypothesis* 3: Considering students' recovery and the effectiveness of anxiety reduction, the optimal intervention frequency is three times a week, providing regular stimulation and preventing over fatigue.

*Hypothesis* 4: Considering students' physical and mental conditions and the effectiveness of anxiety reduction, the optimal duration for each intervention is hypothesized to be 50 minutes.

*Hypothesis* 5: We assume that an 8 week duration for the whole exercise intervention would yield the best results.

## Methodology

The meta-analysis has been pre-registered. The registration website is the Systematic Review and Meta-analysis Registration platform at https://inplasy.com/, and the identifier is INPLASY2024110006.

### Acquisition and preliminary screening of literature

Literature searches were conducted across major Chinese and English databases on October 23, 2024. The search encompassed the China National Knowledge Infrastructure (CNKI) for Chinse literature, and Web of Science, PubMed, Medline, Embase, and Scopus, for English publications. The search period covered all available literature from the inception of each database through October 23, 2024.

The search strategy employed two groups of keywords for both Chinese and English databases, combined using Boolean operators. For Chinese databases, the first keyword group included: “exercise,” “tai chi,” “yoga,” “qigong,” “resistance,” “physical activity,” “movement,” “Baduanjin,” “Pilates,” and “aerobic exercise.” The second group comprised “anxiety” and “mental health.” For English databases, the first keyword group consisted of “exercises,” “physical exercise,” “physical activity,” “aerobic exercise,” “isometric exercise,” “acute exercise,” and “exercise training.” The second group included “anxiety,” “angst,” “nervousness,” “hypervigilance,” “social anxiety,” “anxiety social,” “social anxieties,” and “anxiousness.”

Keywords within each group were combined using the Boolean operator “OR,” while the two groups were connected using “AND.” the complete search strategy was first developed for PubMed and subsequently adapted for other databases. The search query used in PubMed is as follow

(“Exercise”[MeSH Terms] OR “physical exercise”[Title/Abstract] OR “physical activity”[Title/Abstract] OR “aerobic exercise”[Title/Abstract] OR “isometric exercise”[Title/Abstract] OR “acute exercise”[Title/Abstract] OR “exercise training”[Title/Abstract]) AND (“Anxiety”[MeSH Terms] OR “Angst”[Title/Abstract] OR “Nervousness”[Title/Abstract] OR “Hypervigilance”[Title/Abstract] OR “social anxiety”[Title/Abstract] OR “anxiety social”[Title/Abstract] OR “social anxieties”[Title/Abstract] OR “Anxiousness”[Title/Abstract]) AND “college students”[Title/Abstract].

Meanwhile, the search query of Web of science is as follow:

1 (((((TS = (Anxiety)) OR TS = (Angst)) OR TS = (Nervousness)) OR TS = (Hypervigilance)) OR TS = (social anxiety)) OR TS = (Anxiousness).

2 (((((((TS = (Exercise)) OR TS = (physical exercise)) OR TS = (physical activity)) OR TS = (aerobic exercise)) OR TS = (isometric exercise)) OR TS = (acute exercise)) OR TS = (exercise training)).

3 TS = (college students).

#1AND #2 AND #3.

### Inclusion and exclusion criteria of literature

The literature selection process followed the PICOS framework of evidence-based medicine, which systematically evaluates research subjects (P), intervention measures (I), control/comparison measures (C), outcome indicators (O), and study design (S; Liberati et al., 2009). Studies were included if they employed randomized controlled trial (RCT) design, recruited college students as participants, and included samples meeting established anxiety symptom diagnostic criteria. The interventions needed to implement mind–body integrated exercises such as Pilates, Tai Chi, yoga, or Baduanjin, with sessions lasting at least 30 min and conducted multiple times over the intervention period. Studies were required to utilize non-exercise activities or maintenance of regular lifestyle as control conditions and apply validated subjective scales for anxiety assessment. Additionally, studies needed to report complete sample sizes, means, and standard deviations for both experimental and control groups.

Studies were excluded if they were conference abstracts, case studies, or systematic reviews. The exclusion criteria also encompassed studies that included participants with regular physical exercise experience exceeding 3 months or employed cognitive therapy as a control condition. Studies with incomplete pre- or post-intervention data or those published in languages other than Chinese or English were also excluded from the analysis.

### Literature screening and data extraction

**Figure 1:**
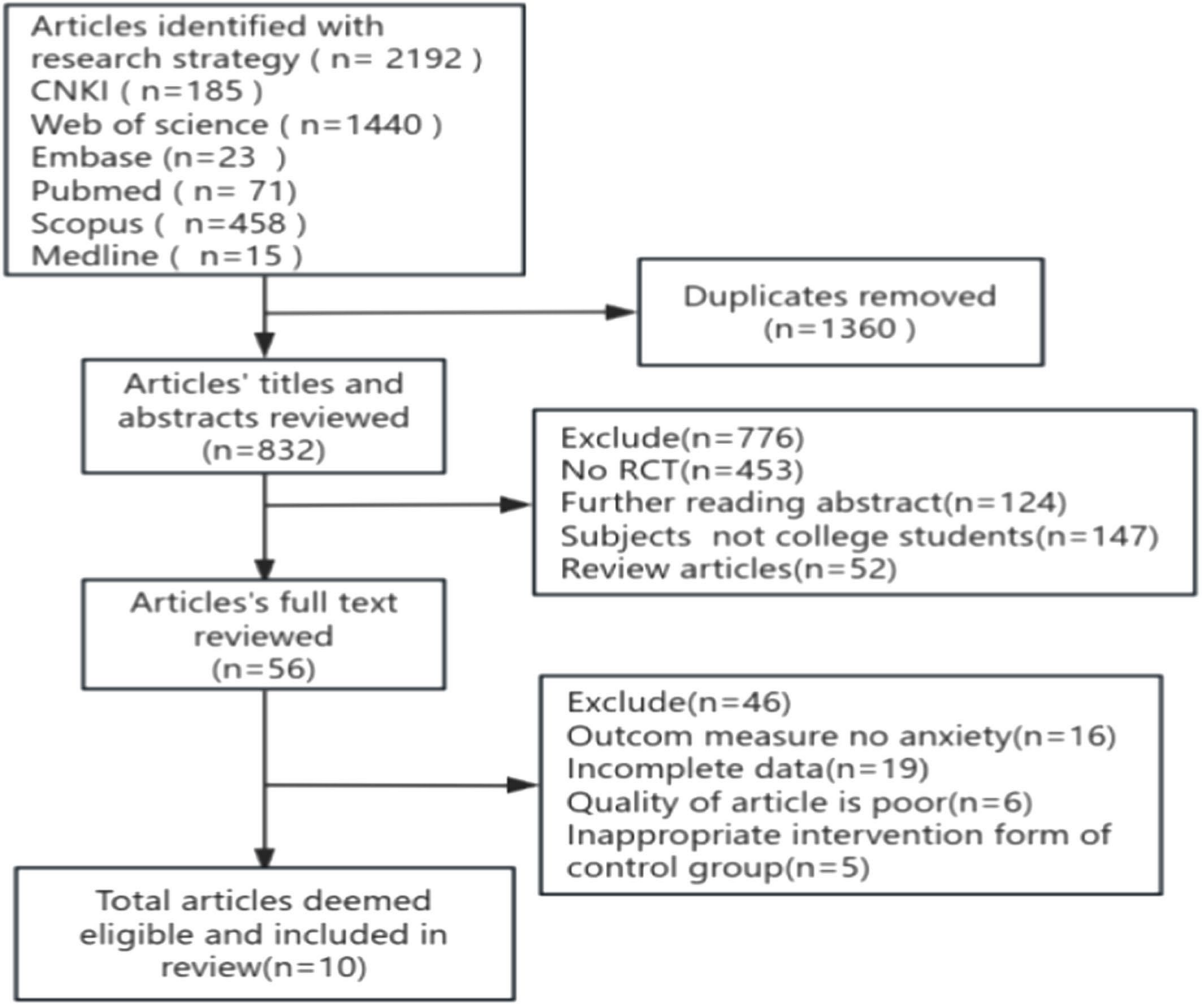
Flow diagram of literature selection.

The literature screening process followed a systematic, multi-step approach. Initially, all identified bibliographic entries were imported into NoteExpress reference management software for duplicate removal. The first author conducted preliminary screening based on titles, abstract, and full text of the publication. Subsequently, a second author independently assessed the selected articles for adherence to the inclusion criteria. In cases where consensus could not be reached, a third author was consulted, or the research team engaged in collaborative discussions to resolve discrepancies. Through this rigorous screening process, the final set of studies for meta-analysis was determined (Figure 1).

Data extraction was conducted independently by two researchers following a standardized protocol. Key indicators extracted included the first author, publication year, sample characteristics (size and gender distribution) and detailed intervention parameters (content, total duration, weekly frequency, and session duration), along with outcome assessment measures. When encountering incomplete or unclear data, the researchers systematically contacted the corresponding authors via email. A follow-up email was sent after 2 weeks if no initial response was received. Articles with persistently missing or ambiguous data after these communication attempts were excluded from the final analysis.

### Literature review quality assessment

The methodological quality of included studies was evaluated using the Physiotherapy Evidence Database (PEDro) scale. The PEDro scale comprises 11 items, with the first item excluded from scoring calculations. Each remaining item was scored dichotomously: 1 point for meeting specified criteria and 0 for not meeting criteria. Studies scoring 6 or higher were classified as high quality. Two researchers independently assessed the included articles using these criteria. When scoring discrepancies arose, resolution was achieved through either consultation with a third researcher or group consensus discussions (Table 1).

**Table 1:** PEDro score of the included literature.

Authors and year of publication	Eligibility criteria specified	Random allocation	Allocation concealment	Group similar at baseline	Blind subjects	Blind therapists	Blind assessors	Adequate follow-up	Intention-to- treat analysis	Intergroup statistical report	Point estimates and variability measures reported	PEDro score
Abavisani et al. (2019)	1	1	0	1	0	0	0	1	1	1	1	6
Kang (2018)	1	1	0	1	0	0	0	1	1	1	1	6
Tsai et al. (2003)	1	1	0	1	0	0	0	1	1	1	1	6
Zhang and Jiang (2023)	1	1	0	1	0	1	1	1	1	1	1	8
Zhang et al. (2023)	1	1	1	1	0	0	1	1	1	1	1	8
Li et al. (2022)	1	1	0	1	0	0	0	1	1	1	1	6
Sun et al. (2024)	1	1	0	1	1	0	0	1	1	1	1	7
Falsafi (2016)	1	1	1	1	0	0	0	0	1	1	1	6
Albracht-Schulte and Robert-McComb (2018)	1	1	0	1	0	0	0	1	1	1	1	6
Yujing et al. (2019)	1	1	0	1	0	0	0	1	1	1	1	6

### Data analysis

The analysis of anxiety scale outcomes across selected studies was conducted using the random model in Revman 5.3 software. As the outcome measures were continue variables using consistent units, the standardized mean difference (SMD) was selected as the effect size metric. Effect sizes were interpreted according to (Cohen et al., 1983) guideline: SMD <0.2 indicating negligible effect, 0.2 ≤ SMD < 0.5 representing small effect, 0.5 ≤ SMD < 0.8 denoting medium effect, and SMD ≥ 0.8 signifying large effect.

Study heterogeneity was evaluated using the I^2^ statistic. An I^2^ value of zero indicates homogeneity among studies, warranting the use of a fixed-effects model for effect size aggregation. Conversely, I^2^ values ≥50% suggest substantial heterogeneity, necessitating the application of a random-effects model and subsequent subgroup analyses to explore potential sources of variation.

## Results

### The basic characteristics of incorporated research literature

This meta-analysis incorporated 10 randomized controlled trials conducted across various international locations. The total sample comprised 514 college students, with five studies including exclusively female participants and five including both genders. Of the total sample, 67 were males and 385 were females (gender distribution was unreported in one study). Individual study sample sizes ranged from 18 to 78 participants.

The exercise interventions encompassed various modalities including yoga, Tai Chi, Qigong, Pilates, and resistance training. Session duration ranged from 30 to 75 min, with 60-min sessions being most prevalent. Intervention frequency varied from two to five sessions weekly, with three sessions per week being the most common protocol. The total intervention duration spanned from 4 to 16 weeks, with 8-week programs predominating. Control group conditions typically involved maintenance of regular lifestyle patterns without structured physical exercise. Anxiety levels were assessed using standardized instruments including the ATAI, SAS, SCL-90, and HAHA scales (Table 2).

**Table 2:** Basic information of included literature.

Authors and publication years	Number E C	Male and female	Intervention measures E C	Time, frequency, duration	Outcomes	Main findings
Abavisani et al. (2019)	31 31	Not referred to	Pilates keep a regular life	60 min 2times/week 8 weeks	STAI	Before intervention, anxiety scores were 52.74 ± 7.41 (experimental) and 52.48 ± 7.27 (control). After, they were 49.63 ± 7.39 and 51.81 ± 7.16. Study shows Pilates helps reduce medical emergency students’ anxiety.
Kang (2018)	30 30	0 60	Pilates keep a regular life	60 min 3times/week 16 weeks	SCL-90	After Pilates training, the anxiety test scores of female college students in the experimental group were significantly lower than those in the control group.
Tsai et al. (2003)	37 39	38 38	Taijiquan sedentary living	50 min 3times/week 12 weeks	STAI	After 12 weeks of Taijiquan training, as evaluated by using the State–Trait Anxiety Inventory (STAI), both trait anxiety and state anxiety of the experimental group decreased. Taijiquan exercise training can improve the anxiety state of the subjects.
Zhang et al. (2023)	9 9	5 13	Taijiquan without any exercise intervention	60 min 5times/week 8 weeks	SAS	BWTC may relieve anxiety and depression by separately regulating Frontal_Mid_L and Frontal_Mid_Orb_R activities.
Zhang and Jiang (2023)	39 39	0 78	Qigong baduanjin maintain the original lifestyle	60 min 3times/week 12 weeks	SCL-90	After the 12 week intervention, the anxiety score and phobic anxiety score in the Baduanjin group decreased significantly when compared to those in the control group.
Sun et al. (2024)	18 19	18 19	Qigong no regular exercise was conducted.	60 min 5times/week 12 weeks	HAMA	Those in the 3 month Qigong exercise intervention had a significant drop in anxiety, especially mental anxiety.
Li et al. (2022)	13 14	0 27	Cluster resistance training not participating in other structured exercise interventions	50 min 2times/week 8 weeks	SAS	After the intervention, SAS score of the intervention group was significantly decreased (*p* < 0.05)
Falsafi (2016)	23 23	6 40	Yoga without any regular physical activity	75 min __ 8 weeks	HAMA	In the yoga intervention group, anxiety symptoms significantly decreased from the baseline state to the follow-up stage (*p* < 0.01).
Albracht-Schulte and Robert-McComb (2018)	20 20	0 40	Yoga stimulation by emotional images	30 min 2times/week 4 weeks	STAI	For the yoga group, state anxiety significantly decreased from the baseline level to the state after the intervention (*p* = 0.001), but it returned to the baseline values after being exposed to emotional stimuli (*p* < 0.001).
Yujing et al. (2019)	34 36	0 70	Yoga combined with aerobic exercise absence of any regular exercise	40 min 3 times/week 8 weeks	SAS	After exercise intervention, the experimental group’s anxiety score improved significantly from (53.76\pm2.70) to (36.41\pm7.32) (*p* < 0.01), while the control group showed no significant change (*p* > 0.05).

E, Experimental group; C, control group; STAI, State–Trait Anxiety Inventory; SCL-90, Symptom Checklist 90, SAS, Self- rating anxiety scale; HAMA, Hamilton Anxiety Scale.

### Publication bias test

**Figure 2:**
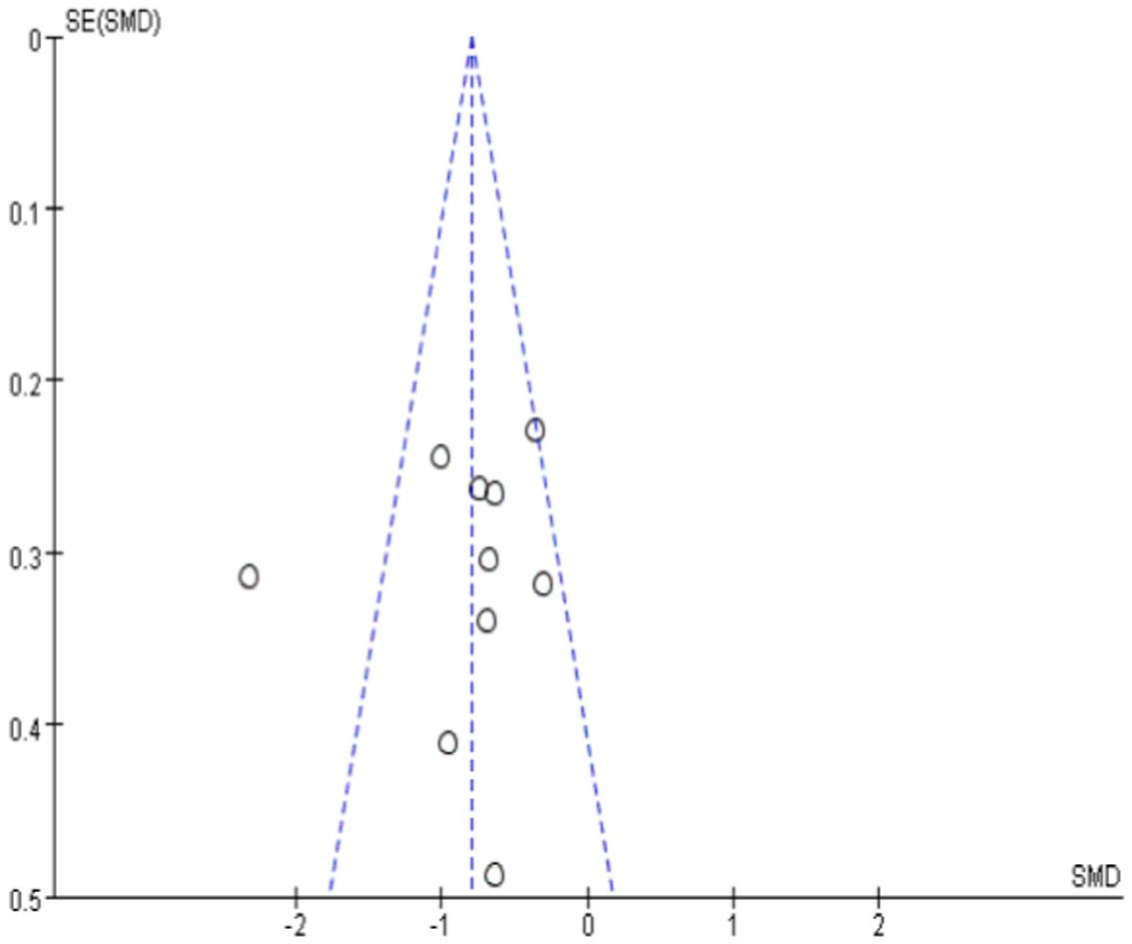
Funnel plots of publication bias.

**Figure 3:**
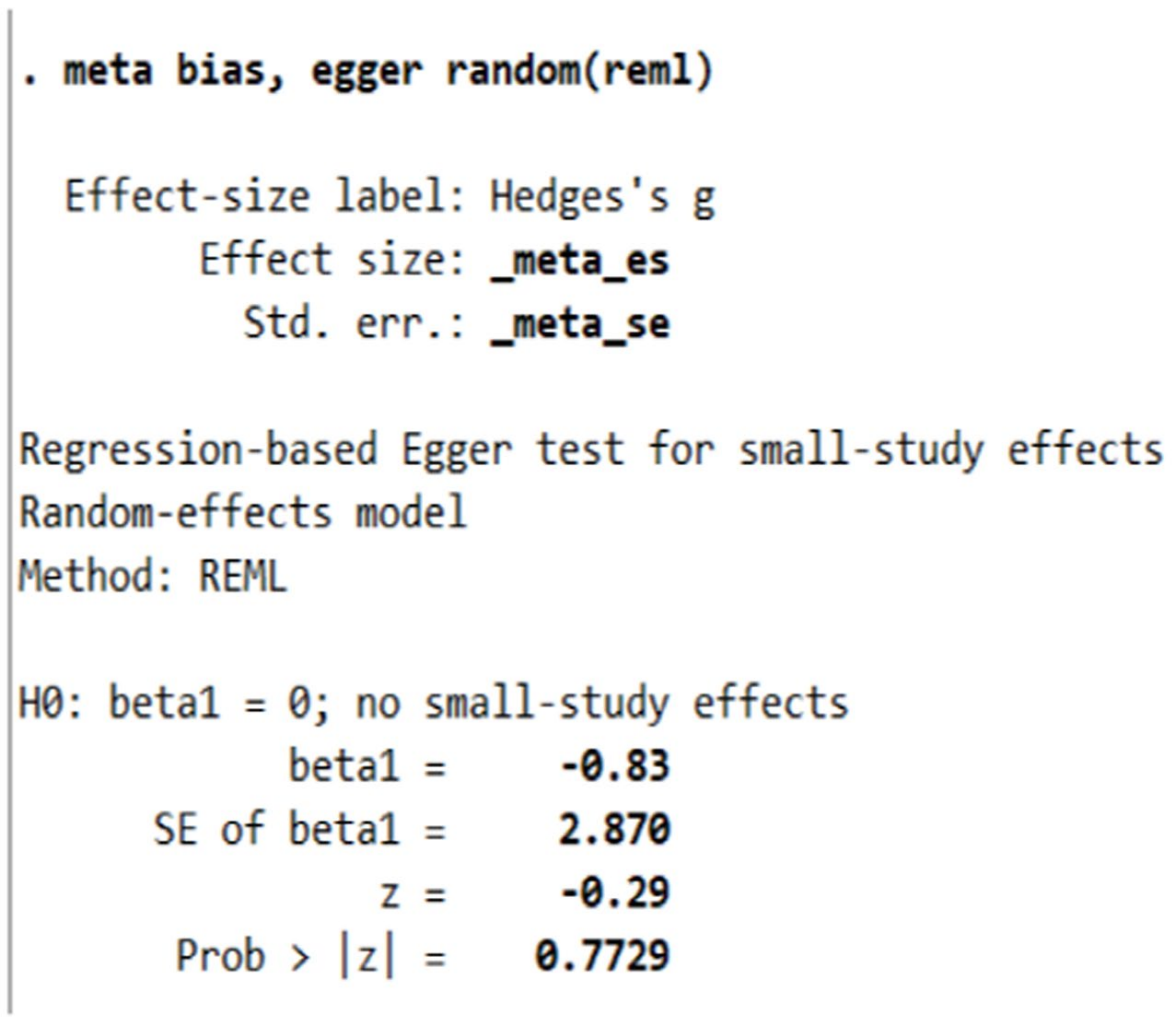
Egger test of publication bias.

Publication bias was assessed through both visual and statistical methods. The funnel plot analysis (Figure 2) revealed one study deviating notably from others, indicating potential heterogeneity in the dataset. The observed asymmetry in the distribution of data points could be attributed to several methodological factors. First, the diversity of intervention methods may contribute to the dispersion of effect sizes. Second, the variation in anxiety measurement instruments across studies, including differences in measurement range, sensitivity, reliability, and validity, could have influenced the symmetry of effect size distribution. For example, studies utilizing State Anxiety Scales versus Trait Anxiety Scales may have captured different aspects of anxiety, potentially contributing to the uneven distribution of effect sizes. Additionally, studies with smaller sample sizes may have produced more extreme effect sizes due to random variation, resulting in asymmetric scatter patterns. However, the Egger test yielded a non-significant result (*p* > 0.05), suggesting the absence of significant publication bias (Figure 3). Given the superior reliability of the Egger test compared to funnel plot visual inspection, we primarily based our conclusions regarding publication bias on the statistical test results.

### Sensitivity analysis

Sensitivity analysis was conducted to evaluate the stability and reliability of the meta-analytic findings. The analyses employed two primary approaches: sequential single-study exclusion and model specification variation. Following each modification, effect sizes were recalculated and tested. The results demonstrated minimal fluctuation across there analyses, indicating robust and reliable meta-analytic findings. This stability across different analytical approaches supports the credibility of the study’s conclusions.

### Meta-analysis results

#### Overall effect test of intervention outcomes

Analysis of the aggregated data from the 10 selected studies revealed a significant effect of physical excise interventions on anxiety symptoms among college students (Table 3). The heterogeneity assessment yielded substantial variation. The heterogeneity assessment yielded substantial variation across studies (I^2^ = 72%, *p* < 0.001), necessitating the use of a random effects model for effect size synthesis. The considerable heterogeneity observed suggests that the intervention effects may be moderated by various factors, warranting further investigation of potential moderating variables that influence the overall treatment efficacy.

**Table 3:** Overall effect of exercise intervention on anxiety.

Quantity of literature	Heterogeneity test			Effect size and 95% confidence interval	Two-tailed test	
	*X^2^*	*p*	*I^2^*		*Z*	*p*
10	31.60	<0.001	72%	−0.83(−1.18, −0.48)	4.62	<0.001

**Figure 4:**
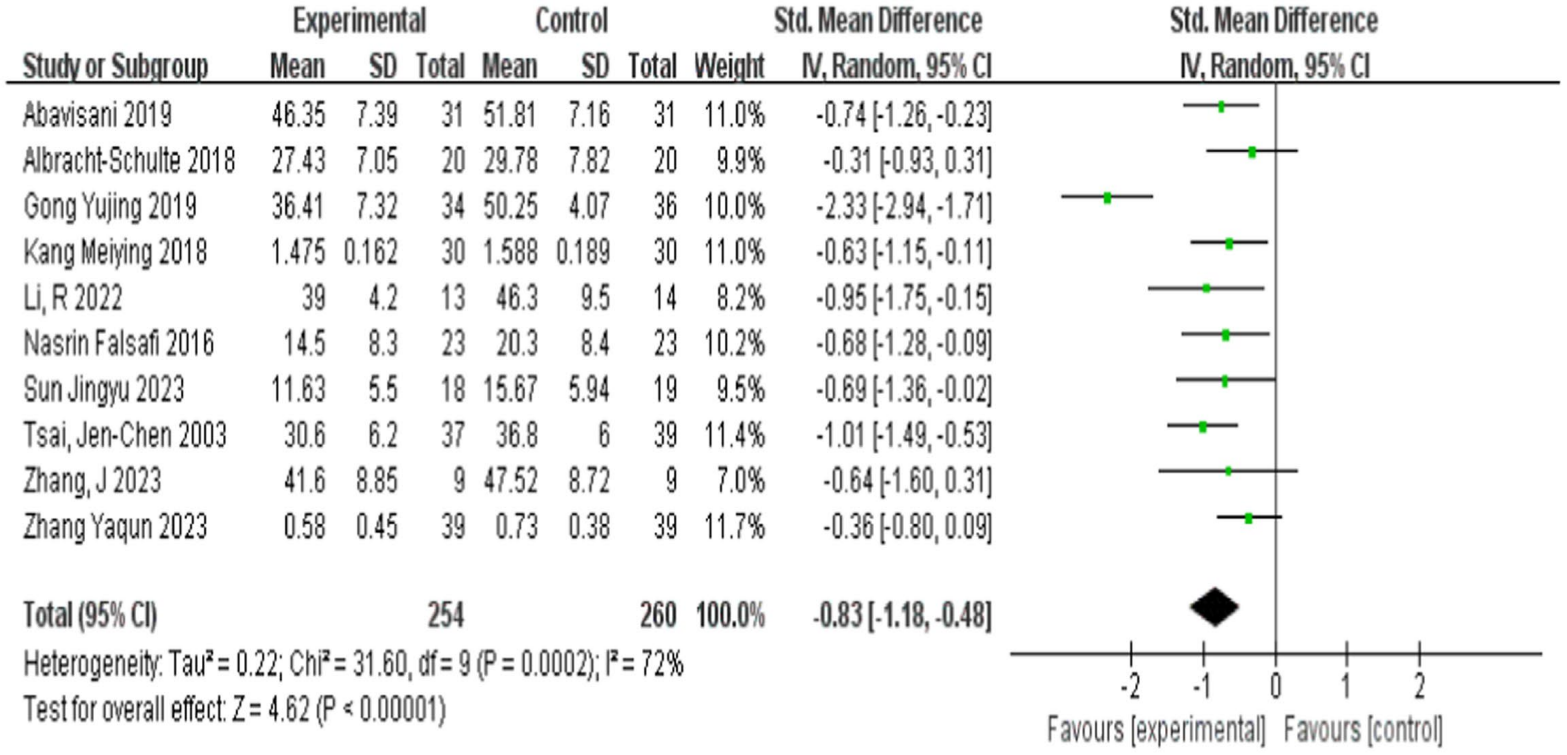
Forest graph of exercise improves the overall effect of anxiety.

The meta-analysis yielded a significant negative effect size (*d* = −0.83, 95% CI [−1.18, −0.48], *p* < 0.001), indicating a substantial reduction in anxiety symptoms following physical exercise interventions among college students (Figure 4). The negative direction of the effect size represents improvement in anxiety symptoms, while the magnitude exceeds Cohen’s (1988) threshold of 0.8 for large effects. The statistical significance (*p* < 0.001) and confidence interval bounds remaining consistently negative provide strong evidence for the robust anxiolytic effects of physical exercise interventions in the college student population. Thus, Research Hypothesis 1 has been verified.

#### Subgroup analysis of moderating variables

Given the substantial heterogeneity observed in the overall effect size analysis, subgroup analyses were conducted to investigate potential moderating variables. Four intervention parameters were examined as potential sources of heterogeneity: intervention measures, weekly frequency, session duration, and total intervention period (Table 4). These analyses aimed to systematically evaluate how variations in exercise program characteristics might influence intervention effectiveness.

**Table 4:** Results of moderating variable intervention on anxiety in exercise program.

Moderating variable	Test of heterogeneity			Subgroup	Quantity of literature	Sample size	Effect size and 95% confidence interval	Two-tailed test	
	*X^2^*	*p*	*I^2^*					*Z*	*p*
Intervention measures	0.08	0.77	0%	Pilates	2	122	−0.69(−1.05, −0.32)	3.68	*p* < 0.001
	0.66	0.42	0%	Qigong	2	115	−0.46(−0.83, −0.09)	2.43	0.02
	0.45	0.50	0%	Taijiquan	2	94	−0.93(−1.36, −0.51)	4.27	*p* < 0.001
	0.72	0.40	0%	yoga	2	86	−0.50(−0.94, −0.07)	2.29	0.02
Intervention Frequency	1.80	0.41	0%	2times/week	3	129	−0.64(−1.00, −0.29)	3.53	*p* < 0.001
	27.58	*p* < 0.001	89%	3times/week	4	284	−1.06(−1.83, −0.29)	2.69	0.007
	0.01	0.94	0%	5times/week	2	55	−0.67(−1.22, −0.13)	2.42	0.02
Duration of a single intervention	20.44	*p* < 0.001	95%	30-40 min	2	110	−1.32(−3.30, 0.66)	1.31	0.19
	0.01	0.91	0%	50 min	2	103	−0.99(−1.40, −0.58)	4.73	*p* < 0.001
	1.49	0.83	0%	60 min	5	255	−0.58(−0.83, −0.33)	4.52	*p* < 0.001
Exercise duration				4 weeks	1	40	−0.31(−0.93, 0.31)	0.97	0.33
	20.14	*p* < 0.001	80%	8 weeks	5	223	−1.09(−1.76, −0.41)	3.17	0.002
	3.78	0.15	47%	12 weeks	3	191	−0.68(−1.09, −0.26)	3.20	0.001
				16 weeks	1	60	−0.63(−1.15, −0.11)	2.39	0.02

**Intervention measures**. The analysis of intervention content included 417 participants across four exercise modalities: Pilates, Qigong, Tai Chi, and Yoga. Notably, the between-group heterogeneity was minimal (I^2^ = 0%), suggesting consistent effects across different exercise types and indicating reliable, predictable intervention outcomes regardless of the specific exercise modality. Among these interventions, Tai Chi demonstrated the largest effect size (*d* = −0.93, 95% CI [−1.36, −0.51], *p* < 0.001), followed by Pilates (*d* = −0.69, 95% CI [−1.05, −0.32], *p* < 0.001). Mind–body exercise programs, specifically Qigong (*d* = −0.46, *p* = 0.02) and Yoga (*d* = −0.50, *p* = 0.02), showed comparable moderate effects. The consistency in anxiety reduction across these diverse exercise modalities, as evidenced by the low heterogeneity, suggests that various forms of structured physical activity may share common therapeutic mechanisms in anxiety management among college students.

**Weekly Intervention Frequency**. Analysis of weekly intervention frequency included 468 participants, categorized in two three frequency levels, two, three and five sessions per week. This analysis revealed substantial heterogeneity among frequency groups (I^2^ = 75%), indicating considerable variability in intervention effects across different frequency protocols. The three-sessions-per-week protocol demonstrated the largest effect size (*d* = −1.06, 95% CI [−1.83, −0.29], *p* = 0.007), followed by the five-sessions-per-week protocol (*d* = −0.67, 95% CI [−1.22, −0.13], *p* = 0.02). The two-sessions-per-week protocol showed the smallest, though still significant, effect (*d* = −0.64, 95% CI [−1.00, −0.29], *p* < 0.001). However, the high heterogeneity suggests that these findings should be interpreted with caution, as the relationship between intervention frequency and anxiety reduction may be influenced by other unmeasured variables.

**Duration of Each Single Intervention**. The analysis of intervention session duration included 468 participants, with substantial heterogeneity observed across duration groups (I^2^ = 75%). This heterogeneity may be attributed to variations in anxiety measurement instruments across studies, with different scales potentially varying in their sensitivity and specificity. The 50-min session protocol demonstrated the strongest anxiolytic effect (*d* = −0.99, 95% CI [−1.40, −0.58], *p* < 0.001), followed by the 60-min session protocol (*d* = −0.58, 95% CI [−0.83, −0.33], *p* < 0.001).

**Exercise Intervention Period**. The analysis of total intervention duration encompassed 514 participants, with substantial heterogeneity observed across duration groups (I^2^ = 72%), indicating that intervention length significantly moderated the relationship between exercise and anxiety reduction. The 8-week intervention protocol demonstrated the largest effect size (*d* = −1.09, 95% CI [−1.76, −0.41], *p* = 0.002), followed by the 12-week protocol (*d* = −0.68, 95% CI [−1.09, −0.26], *p* = 0.001). A declining trend in effectiveness was observed with longer intervention periods, as evidenced by the 16-week protocol (*d* = −0.63, 95% CI [−1.15, −0.11], *p* = 0.02). The 4-week intervention showed the smallest effect size (*d* = −0.31) and failed to reach statistical significance (*p* > 0.05), suggesting that shorter intervention periods may be insufficient for meaningful anxiety reduction.

Up to this point, Hypotheses 1–5 have all been verified.

## Discussion

### Quality of included literature and overall effect size

Quality assessment of the 10 included studies yielded scores ranging from 6 to 8, with a mean score of 6.5, indicating generally high methodological quality. Lower quality scores were primarily attributed to insufficient reporting of randomization procedures, blinding protocols and participant attribution. Publication bias analysis revealed symmetric distribution of studies, suggesting robust stability of findings. The meta-analysis demonstrated a large significant effect of exercise interventions on anxiety reduction among college students (*d* = −0.83, 95% CI [−1.18, −0.48], *p* < 0.001).

### Importance and significance of the research results

This meta-analysis systematically assesses the effectiveness of physical exercise interventions, especially mind–body integrated exercises such as yoga, Pilates, Tai Chi, and Qigong, in alleviating anxiety among college students, and precisely determines the optimal intervention parameters. Recent meta-analyses evaluating the impact of exercise interventions on anxiety in elderly cancer patients demonstrated that exercise was significantly associated with a reduction in anxiety levels (standardized mean difference [SMD] = −0.39; 95% confidence interval [CI], −0.66 to −0.12; Soong et al., 2025). Moreover, subgroup analyses revealed that, in contrast to conventional exercise interventions, mind−body exercise interventions were more strongly associated with improvements in anxiety levels (SMD = −0.77; 95% CI, −1.54 to −0.01; Soong et al., 2025), which is highly consistent with the finding of our study that mind–body exercise can ameliorate anxiety among college students. Furthermore, research has indicated that mind–body exercises (−0.67 [95% confidence interval = −1.19 to −0.15]) and resistance exercises (−1.00 [95% confidence interval = −1.70 to −0.30]) have a significant effect on alleviating anxiety in Parkinson’s disease patients (Costa et al., 2024). This provides crucial theoretical basis and practical support for our research approach, in which mind–body exercises are adopted as almost all the exercise interventions, further validating the scientific nature and forward–looking nature of the research direction. From the overall subgroup analysis, the effects of lower-intensity exercise, lower exercise frequency, and shorter exercise intervention cycles on anxiety were not statistically significant, and the 95% confidence interval included 0 (Lin and Gao, 2023). However, the subgroup analysis of this study shows that the identified optimal intervention protocol is to conduct exercises three times a week, 50 min each time, for a period of 8 weeks, and this protocol has the most significant anxiolytic effect among the college student population. The inconsistency of the research results indicates that when designing exercise interventions aimed at addressing the anxiety problems of college students, it is crucial to conduct in-depth exploration and precisely determine these key parameters in order to maximize the positive impact of exercise on mental health.

As the key force for future social development, the mental health of college students is of crucial importance. However, anxiety is widespread and seriously impacts their academic performance, daily life, and social interactions (Andrews and Wilding, 2004). Mind–body integrated exercises have remarkable advantages in addressing college students’ anxiety. Campuses are equipped with sufficient venues and rich club activities, enabling students to easily participate in their spare time. They can either practice yoga alone to relax or engage in group Tai Chi sessions to relieve stress. Such convenience lowers the participation threshold. College students attach great importance to self-image and quality improvement (Crocker and Canevello, 2012).These exercises can not only relieve anxiety but also enhance physical fitness, shape the body, and achieve dual optimization of the body and mind, which is highly appealing. Moreover, college students have a strong sense of self-control (Bowlin and Baer, 2012).The control of breathing, movements, and mental focus during exercise allows them to truly feel in control of their lives and emotions, strengthening their confidence in coping with anxiety (Otto and Smits, 2011).

This research offers robust empirical evidence for the effectiveness of exercise interventions in alleviating anxiety among college students. Institutions of higher education are advised to augment their investment in sports facilities. This involves constructing specialized yoga and Pilates classrooms, as well as creating well-maintained outdoor spaces dedicated to the practice of Tai Chi and Qigong. Simultaneously, the curriculum system should be diversified by introducing beginner-level, intermediate-level, advanced-level, and specialized courses, so as to accommodate the diverse requirements of students. A collaborative effort between the mental health center and the physical education teaching department is highly recommended. Psychologists should conduct comprehensive assessments of students’ anxiety levels. Physical education instructors, on the basis of students’ physical conditions and individual preferences, should then design customized exercise regimens. For instance, for students with mild anxiety and good flexibility, meditation-centered yoga courses can be recommended; for those with moderate anxiety and relatively weak physical strength, Pilates core-strengthening training programs can be arranged. Subsequently, the intensity and frequency of the exercises should be rationally determined. The two parties should jointly provide continuous follow-up guidance and conduct regular evaluations and adjustments.

### Exploration of the physiological mechanism of exercise intervention on anxiety in college students

The anxiolytic effects of exercise can be attributed to several neurobiological mechanisms. Exercise facilitates the release of key neurotransmitters including serotonin, dopamine, and endorphins in the brain (Mikkelsen et al., 2017). Serotonin, commonly referred to as the “happiness hormone,” plays a crucial role in mood regulation, sleep patterns and appetite control (LaGreca et al., 2021). Dopamine, a reward-associated neurotransmitter, enhances motivation and please responses (Bressan and Crippa, 2005). While endorphins function as natural analgesics and mood regulators, promoting feelings of well-being and relaxation (Hawkes, 1992). The elevated levels of these neurotransmitters contribute significantly to anxiety reduction among college students (Yuan et al., 2018).

Furthermore, sustained physical exercise promotes neuroplasticity through enhanced neurogenesis and neural connectivity, resulting in increased gray and white matter volume and improved brain function (White and Castellano, 2008). Particularly noteworthy is the exercise-induced enhancement of prefrontal cortex activity, a region critical for emotional regulation, decision-making processes, and attentional control (Dixon et al., 2017). These structural and functional neural adaptations strengthen emotional regulation capabilities and reduce anxiety susceptibility (McEwen, 2007).

### Limitations of the research

This meta-analysis acknowledges several methodological limitations that merit attention. Notably, the fact that multiple studies only cover female samples is a prominent issue. The gender disparities exert a pivotal influence on the efficacy of exercise-based interventions. Males and females differ in their physiological responses to exercise, mental states, and access to social support (Kirschbaum et al., 1995).For example, differences in hormone levels lead to disparities in the tolerance and recovery abilities of males and females to exercise intensity (Sheel et al., 2004).When facing anxiety, the exercise motivations and coping strategies of males and females also vary. For example, testosterone in men may enhance muscle strength and endurance during exercise (Vingren et al., 2010), while estrogen in women affects the body’s energy metabolism and stress response (Mauvais-Jarvis et al., 2013).These physiological differences, in turn, influence the way exercise acts on anxiety. Men may be more inclined to relieve anxiety through high-intensity competitive sports, taking advantage of their physical strength and endurance (Salmon, 2001).In contrast, women may prefer low-intensity, rhythmic exercises such as yoga (Ross and Thomas, 2010),and these types of exercises may have different impacts on their mental states. At the psychological level, men and women also cope with anxiety differently. Men tend to internalize their emotions (Simon and Nath, 2004).When facing anxiety, their exercise motivation may be more about distraction or physical self-improvement. In comparison, women are more willing to express their emotions and may regard exercise as a way to socialize while alleviating anxiety (McLean and Anderson, 2009) Conducting research based solely on female samples cannot comprehensively reveal the mechanism of exercise interventions in alleviating anxiety, nor can it easily determine the differences in intervention effectiveness between genders.

Secondly, excluding individuals who have been engaged in exercise for more than 3 months is reasonable from the perspective of research design. Such long-term exercisers may have developed relatively stable exercise habits and psycho-physiological adaptation mechanisms. Their physical functions and psychological states differ significantly from those of ordinary students. However, this approach also brings non-negligible limitations. In terms of the external validity of the research results, this exclusion criterion restricts the scope of generalization of the research conclusions. In reality, a considerable number of students may have long-term exercise experiences. After excluding this group of people, the research results are difficult to represent the true situation of the entire student population, thus affecting the applicability of the results to a broader student group. In addition, this exclusion criterion may, to some extent, affect the internal validity of the study. Although the homogeneity of the sample is improved to a certain degree after excluding long-term exercisers, some key information may also be lost. The impact mechanism of long-term exercise on students’ anxiety levels may differ from that of short-term exercise.

Finally, the relatively short follow-up periods in the included studies present a notable limitation. Given the chronic nature of anxiety symptoms, longer follow-up periods would be necessary to establish the durability of exercise intervention effects (Asmundson et al., 2013). The absence of extended longitudinal data may limit our understanding of the intervention’s long-term efficacy.

### Clinical recommendations

We propose that higher education institutions forge partnerships with neighboring mental health institutions. Regular consultations by psychiatrists at the school are beneficial, especially for students with severe anxiety symptoms (Rones and Hoagwood, 2000). For those diagnosed with anxiety disorders, in addition to exercise interventions, psychiatrists can prescribe suitable medications. Professional clinical assessment scales like HAMA and BAI should be employed to quantitatively evaluate students’ anxiety levels before and after exercise intervention. Regular monitoring of physiological indicators such as heart rate variability and cortisol levels helps objectively reflect the impact of exercise on anxiety (Kim et al., 2018). In case of non-improvement or symptom worsening, the intervention plan must be promptly adjusted, and referral to professional medical institutions should be considered. All these efforts should be coordinated with the school’s mental health center and physical education teaching department to form a comprehensive approach.

### Future research directions

Future research should address methodological rigor through enhanced research designs, larger sample sizes, and standardized measurement protocols to ensure greater reliability and validity of findings. The implementation of unified assessment tools and evaluation criteria would facilitate more robust cross-study comparisons and strengthen the evidence base. Investigation of the underlying mechanisms of exercise-induced anxiety reduction should encompass both physiological and psychological pathways (Tseng et al., 2012). This includes examining the effects of exercise on neural neurotransmitter systems, cardiovascular function, immune response, as well as cognitive processes, emotional regulation, and self-concept development. Such mechanistic understanding would provide a stronger theoretical foundation for exercise-based interventions. Longitudinal studies with extended follow-up periods are essential to evaluate the durability and sustainability of exercise interventions’ anxiolytic effects (Stonerock et al., 2015). These temporal insights would inform the development of more effective long-term mental health intervention strategies in collegiate settings. Finally, future research should explore integrated intervention approaches combining exercise with other therapeutic modalities, such as cognitive-behavioral therapy, psychological counseling, and social support interventions. Such comprehensive intervention protocols may yield enhanced therapeutic outcomes through synergistic effects.

## Conclusion

This meta-analysis demonstrates that exercise interventions significantly reduce anxiety symptoms among college students. Among various mind–body integrative exercise modalities, Tai Chi emerged as particularly effective in anxiety reduction. The optimal intervention protocol identified consists of three 50-min sessions per week over an eight-week period, yielding the most substantial anxiolytic effects in the collegiate population.

From a theoretical perspective, this study advances our understanding of exercise-based anxiety interventions by establishing clear dose–response relationships and identifying the relative efficacy of different exercise modalities. These findings contribute to the broader literature on non-pharmacological anxiety management and provide a framework for understanding how specific exercise parameters influence mental health outcomes in young adults. The identification of optimal intervention characteristics offers valuable insights into the mechanisms through which physical activity influences psychological well-being.

From a practical standpoint, these findings provide evidence-based guidelines for implementing exercise-based mental health interventions in higher education settings. University health services and counseling centers can utilize these results to design and implement structured exercise programs as part of their mental health support services. The clear delineation of optimal frequency, duration, and type of exercise enables institutions to develop targeted, efficient interventions that maximize therapeutic benefits while considering resource constraints and student schedules. Furthermore, these findings support the integration of structured physical activity programs into comprehensive student mental health strategies, offering a cost-effective, accessible, and stigma-free approach to anxiety management in collegiate populations.

## Data Availability

The original contributions presented in the study are included in the article/supplementary material, further inquiries can be directed to the corresponding author.
